# Genome-scale hypomethylation in the cord blood DNAs associated with early onset preeclampsia

**DOI:** 10.1186/s13148-015-0052-x

**Published:** 2015-03-13

**Authors:** Travers Ching, James Ha, Min-Ae Song, Maarit Tiirikainen, Janos Molnar, Marla J Berry, Dena Towner, Lana X Garmire

**Affiliations:** Molecular Bioscience and Bioengineering Graduate Program, University of Hawaii at Manoa, Honolulu, HI 96822 USA; Epidemiology Program, University of Hawaii Cancer Center, Honolulu, HI 96813 USA; Division of Biology and Biological Engineering, California Institute of Technology, Pasadena, CA 91126 USA; Epidemiology Department, The Ohio State University, The College of Public Health, Columbus, OH 43210 USA; University of Hawaii Cancer Center, The Ohio State University and James Cancer Hospital, Columbus, OH 43210 USA; Genomics Shared Resources, University of Hawaii Cancer Center, Honolulu, HI 96813 USA; Department of Cell and Molecular Biology, John A. Burns School of Medicine, University of Hawaii, Honolulu, HI 96813 USA; Department of Obstetrics, Gynecology and Women’s Health, John A. Burns School of Medicine, University of Hawaii, Honolulu, HI 96826 USA

**Keywords:** Preeclampsia, Epigenetics, DNA methylation, Cord blood, Bioinformatics

## Abstract

**Background:**

Preeclampsia is one of the leading causes of fetal and maternal morbidity and mortality worldwide. Preterm babies of mothers with early onset preeclampsia (EOPE) are at higher risks for various diseases later on in life, including cardiovascular diseases. We hypothesized that genome-wide epigenetic alterations occur in cord blood DNAs in association with EOPE and conducted a case control study to compare the genome-scale methylome differences in cord blood DNAs between 12 EOPE-associated and 8 normal births.

**Results:**

Bioinformatics analysis of methylation data from the Infinium HumanMethylation450 BeadChip shows a genome-scale hypomethylation pattern in EOPE, with 51,486 hypomethylated CpG sites and 12,563 hypermethylated sites (adjusted *P <*0.05). A similar trend also exists in the proximal promoters (TSS200) associated with protein-coding genes. Using summary statistics on the CpG sites in TSS200 regions, promoters of 643 and 389 genes are hypomethylated and hypermethylated, respectively. Promoter-based differential methylation (DM) analysis reveals that genes in the farnesoid X receptor and liver X receptor (FXR/LXR) pathway are enriched, indicating dysfunction of lipid metabolism in cord blood cells. Additional biological functional alterations involve inflammation, cell growth, and hematological system development. A two-way ANOVA analysis among coupled cord blood and amniotic membrane samples shows that a group of genes involved in inflammation, lipid metabolism, and proliferation are persistently differentially methylated in both tissues, including *IL12B*, *FAS*, *PIK31*, and *IGF1*.

**Conclusions:**

These findings provide, for the first time, evidence of prominent genome-scale DNA methylation modifications in cord blood DNAs associated with EOPE. They may suggest a connection between inflammation and lipid dysregulation in EOPE-associated newborns and a higher risk of cardiovascular diseases later in adulthood.

**Electronic supplementary material:**

The online version of this article (doi:10.1186/s13148-015-0052-x) contains supplementary material, which is available to authorized users.

## Background

Preeclampsia is a pregnancy-specific disease characterized by hypertension (blood pressure >140/90 mmHg) and proteinuria (>0.3 g/day). It affects 5% to 8% of all pregnancies [1] and is one of the leading causes of fetal/neonatal and maternal morbidity and mortality [2]. Preeclampsia may develop at any time after 20 weeks of pregnancy, and depending on the time of the onset of symptoms (earlier or later than 34 weeks), it can be categorized as early onset preeclampsia (EOPE) or late onset preeclampsia (LOPE). Although EOPE and LOPE share some clinical symptoms, they have distinct etiologies, biochemical markers, and maternal and fetal outcomes [3]. EOPE is considered a more severe, mostly fetal disorder, and it is typically associated with inadequate placental implantation and subsequent placental dysfunction, intrauterine growth restriction, low birth weight, and adverse maternal and fetal outcomes, including perinatal death. By contrast, LOPE is considered a maternal disorder often associated with a normal placenta, larger placental volume, normal fetal growth, and favorable maternal and fetal outcomes. In this study, we focus on EOPE given its severity.

The etiology of EOPE is unknown. It is hypothesized that the process is initiated by the shallow implantation of the placenta into the uterine wall [4]. During normal pregnancy, extravillous trophoblasts remodel the spiral arteries in the decidua lining during the formation of the maternal placenta. However, in EOPE, such remodeling is incomplete, leading to poor placental development and function [5]. The incompletely transformed spiral arteries are unable to provide an adequate blood supply to the placenta, resulting in ischemia and hypoxic conditions, which further lead to inflammatory stress, apoptosis, and other phenomena in the trophoblasts [6-8]. In mothers, the pathological manifestations of these conditions include hypertension, thrombocytopenia, hemolysis, seizures, acute atherosis, renal failure, and proteinuria [9-11]. Whereas in the fetus, the consequences include intrauterine growth restriction and preterm delivery with associated issues such as neurodevelopmental disorders [12] and long-term adult onset disorders including diabetes, congestive heart failure, and hypertension [13].

Increasing evidence shows that epigenetic processes, especially DNA methylation, play important roles in preeclampsia [14]. While the majority of epigenetic studies have been focusing on detecting differentially methylated regions in the maternal blood [15,16] or placental tissues [17-20], only a few studies have investigated the alteration of local or global methylation and epigenetic factors in neonatal cord blood cells associated with preeclampsia. At the individual gene level, *IGF2* but not *GNAS* has been found to be significantly hypomethylated in cord blood cells associated with maternal preeclampsia [21]. At the global level, controversy exists regarding the plausible relationship between DNA methylation and preterm birth, a major consequence of maternal EOPE. While some authors have proposed that preeclampsia might epigenetically program placental tissue but not umbilical cord blood [22], others have found that preterm birth is associated with overall lower LINE-1 methylation levels in cord blood, which could be a surrogate of global DNA methylation levels [23]. However, to our knowledge, there has been no epigenome-scale methylation study on cord blood DNAs in association with EOPE. In this study, we have for the first time used the Infinium Human Methylation 450 K BeadChip (450 K) to quantitatively analyze methylation on over 485,000 CpG sites in the cord blood DNAs from EOPE mothers (cases) and full-term controls. Our bioinformatics and systems biology analyses have revealed a global hypomethylation pattern in DNA extracted from cord blood in infants born at <34 weeks due to EOPE and have found many changes related to immune response and cardiovascular dysfunction. The discovery could shed light on the connection to adult-onset diseases in the newborns from EOPE mothers.

## Results

### Global CpG hypomethylation in cord blood DNAs associated with maternal EOPE

We compared the genome-scale methylation profile of 20 cord blood DNAs associated with maternal EOPE cases and normal term delivery controls (12 cases vs. 8 controls). A summary of physiological and clinical factors associated with the maternal samples is tabulated in Table 1, including mother’s age, gestational age, gravida, parity, baby gender, and birth weight. We did not observe statistical differences in maternal age, gravida, parity, and sex of babies between cases and controls. As a consequence of EOPE, the patients had to deliver the babies prematurely to terminate the potentially fatal EOPE condition. Thus, both gestational age and mean weight of the babies were significantly different between EOPE and full-term controls (31.5 weeks versus 39.3 weeks, *P =* 2.28e − 8 and 1,350 g versus *P =* 1.17e − 9 and 3,420 g, respectively).

**Table 1:** **The physiological and clinical parameters of study subjects**

**Parameters**	**Case**	**Control**	***P*** **value**
Mother’s age (years)	30.5 ± 6.15	29.6 ± 5.15	0.7352
Gestational age (weeks)*	31.5 ± 2.15	39.3 ± 0.49	2.28e − 8
Gravida	2.33 ± 1.78	2.75 ± 2.38	0.6794
Parity	1.00 ± 1.13	1.12 ± 1.64	0.8544
Baby’s weight (grams)*	1,350 ± 400	3,420 ± 352	1.17e − 09
Gender	5 Female/7 male	5 Female/3 male	0.4476084

*P < 0.05.

**Figure 1:**
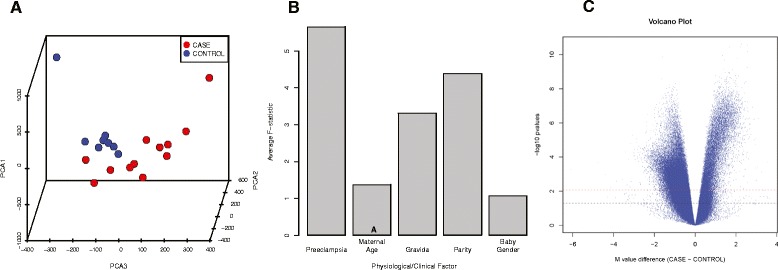
**Global CpG hypomethylation in cord blood cells is associated with maternal preeclampsia. (A)** 3D Principal Components Analysis (PCA) of the methylation M-values from cord blood samples. Preeclampsia-associated cases are shown in red, while controls are in blue; PCA1, PCA2, and PCA3 denote principal components 1, 2, and 3, respectively. The two groups are clearly separated when viewed using the first three principal components. **(B)** ANOVA plot of clinical factors using the methylation M-values in cord blood samples. Averaged ANOVA F-statistics are calculated for potential confounding factors, including preeclampsia, maternal age, gravida, parity, and baby gender. **(C)** Volcano plot of all CpG methylation M-values. The *y*-axis of the negative log10 transformation of the *P* values obtained from differential methylation analysis of the CpG sites is plotted against the *x*-axis of the difference in the average M-values between cases and controls. The dashed red line marks the *α* = 0.05 significance threshold after Benjamini-Hochberg correction of multiple hypothesis tests (MHT), whereas the dashed black line marks the unadjusted *α* = 0.05 significance threshold.

We first performed Principal Component Analysis (PCA) with the M-values of the 384,632 pre-processed methylation data points (Figure 1A). The cases were separable from the controls with the first three principal components, indicating that the cord blood cell methylation data provide strong signatures to the maternal preeclampsia condition. To systematically determine how the physiological and clinical factors contributed to variance in the data, we performed analysis of variance (ANOVA) and computed the average F-statistic for each factor (Figure 1B). Given that gestational age and baby weight differences are the direct consequences of EOPE and thus they are highly correlated to EOPE, we use the term “preeclampsia” to represent the overall phenotype of these three factors. ANOVA analysis shows that preeclampsia condition is the highest source of variance, followed by parity and gravida. To determine the best statistical model for the differential methylation (DM) test, we used Akaike’s information criterion (AIC) to compare the effects of physiological and clinical factors on the linear model. The result shows that the model with just preeclampsia had the best score and was selected more often than the model containing parity and/or gravida. We therefore conclude that maternal preeclampsia is the primary source for the differences in methylation among samples.

**Figure 2:**
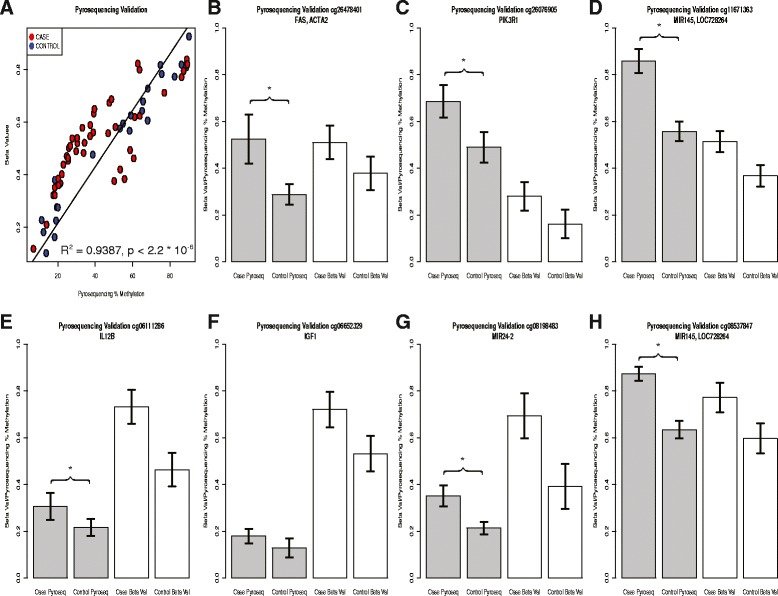
**Technical validation of a set of DM CpG sites using pyrosequencing. (A)** The overall correlation plot between the Beta values of seven DM CpG sites obtained from Illumina HumanMethylation450 BeadChip array (*y*-axis) and methylation percentage from pyrosequencing (*x*-axis). Five case and five control samples for each CpG site were selected for pyrosequencing validation. **(B, C, D, E, F, G, H)** Bar plots to compare the average Beta values and pyrosequencing methylation percentage for individual CpG sites in **(A)**: **(B)** cg26478401, **(C)** cg26076905, **(D)** cg11671363, **(E)** cg06111285, **(F)** cg06652329, **(G)** cg08198483, and **(H)** cg08537847. Values are presented as mean ± standard deviation (s.d.). The CpG sites that have significant *P* < 0.05 between cases and controls are labeled by (*).

The DM analysis using a moderated *t*-test identified a total of 64,049 significant CpG sites after the Benjamini-Hochberg (BH) multiple hypothesis testing correction with a threshold of *P <*0.05. A global hypomethylation pattern was observed, with 51,486 hypomethylated sites and 12,563 hypermethylated sites (Figure 1C). To validate the results of the Illumina 450 K array, we performed pyrosequencing on seven selected CpG methylation sites (cg26478401 from FAS, cg2607695 from *PI3KR1*, cg06111286 from *IL12B*, cg06652329 from *IGF1*, cg08198483 from *miR-24-2*, cg11671363 and cg08537847 from *miR-145*). A coefficient of correlation (*R*^2^) as high as 0.9387 (*P <* 2.2e − 6) was obtained by comparing the methylation percentages determined by pyrosequencing and the β-values determined by Illumina 450 K (Figure 2A). For individual CpG sites, the directions of fold change in the 450 K compared to those of the pyrosequencing were consistent (Figure 2B, C, D, E, F, G, H), and the differences in pyrosequencing results between the cases and controls were significant. These results show the robustness and validity of the 450 K data.

### Genome-scale patterns of differentially methylated CpG sites in CpG islands and surrounding areas

**Figure 3:**
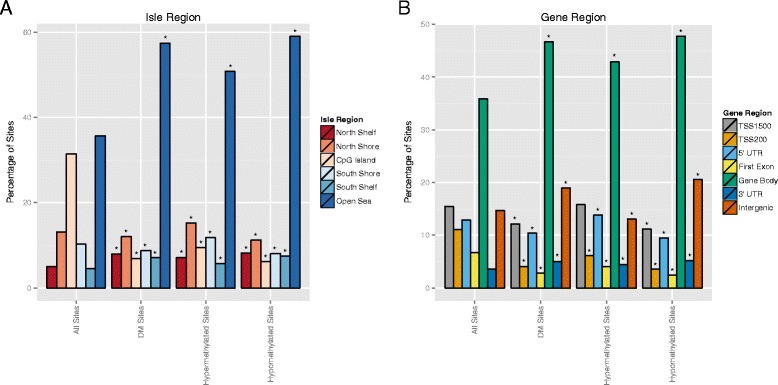
**Comparison of CpG site methylation of cord-blood-cell-associated EOPE and normal mothers.** Distributions of all CpG site categories on the Illumina HumanMethylation450 BeadChip array (background), all differentially methylated CpG sites, hypomethylated CpG sites, and hypermethylated CpG sites. Categories with significantly different proportions (*P <* 0.05, Chi-square test) of differential CpG sites vs. expected background (all CpG sites) are labeled by (*). Differences in categorical proportions between hypomethylation and hypermethylation are labeled the same. **(A)** CpG site proportions by location relative to CpG isle regions. As stated in the “Results” section, six groups are categorized according to Illumina 450 K annotation, namely north shelf, north shore, CpG island, south shore, south shelf, and open sea. **(B)** CpG site proportions by functional location relative to gene regions. Seven groups are categorized, namely the 200 to 1,500 bp upstream of transcription starting site (TSS1500), the transcription starting site to 200 bp upstream interval (TSS200), 5′ UTR, first exon, gene body, 3′ UTR, and the rest for integenic regions. DM, differential methylation.

To examine the methylation profile across genomic locations, we counted the number of DM sites, hypermethylated sites, and hypomethylated sites in different genomic categories relative to CpG islands (Figure 3A). The CpG-related categories are based on the annotation given by Illumina. CpG islands are defined as regions with a GC content of 50% or greater, length greater than 200 bp and with a ratio of observed CpGs to the expected number of CpGs greater than 0.6 [24]. CpG shores are defined as 0 to 2 kb upstream (north) or downstream (south) of the CpG islands and CpG shelves as 2 to 4 kb upstream or downstream of the CpG islands. The open sea regions refer to regions not located in any of the abovementioned categories relating to CpG islands.

**Figure 4:**
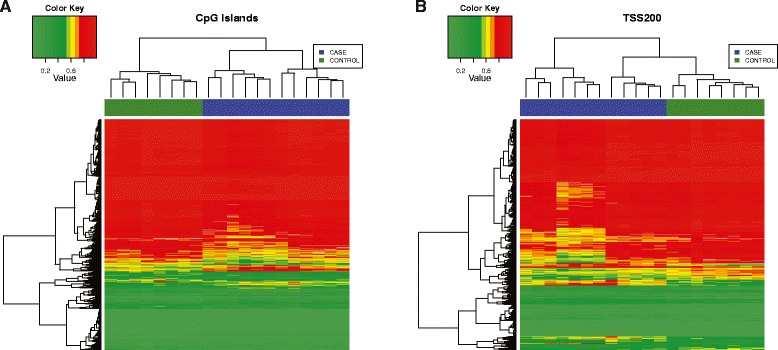
**Heatmaps of DM CpG sites located in CpG islands (A) and the TSS200 region of genes (B).** The rows correspond to CpG sites, while the columns correspond to samples. Case samples are blue, and controls are green. The color of each cell in the heatmap is based on the Beta (*β*) value of the corresponding CpG site in the corresponding sample, with colors ranging from red (*β* = 0) to blue (*β* = 1). The ordering of CpG sites and samples in the heatmaps was determined via unsupervised hierarchical clustering.

Significant differences between observed frequencies and expected frequencies exist in all categories based on CpG-related annotations as stated in the previous paragraph (Figure 3A). The most striking result is the significant (about fourfold) reduction in the percentages of the overall DM and hypermethylated and hypomethylated sites in the CpG islands compared to the background percentage of probes in CpG islands (for example, the percentage of all annotated probes in CpG islands). The hypomethylation pattern is also evident in the heatmap results of CpG islands with unsupervised hierarchical clustering, where the case and control groups are clearly separated (Figure 4A). Conversely, there is significantly more DM, including both hypermethylation and hypomethylation in the open sea region compared to the background percentage (Figure 3A). The majority of CpG islands are associated with gene promoters whereas the open seas are often associated with gene body and intergenic regions. These results show that methylation changes in non-promoter regions of cord blood DNAs are mostly associated with maternal EOPE (Figure 3B).

As examples, we selected the most statistically significant 20 hypomethylated and 20 hypermethylated CpG sites with an average β-value difference greater than 0.2 and present their dot plots in Additional file 1: Figure S1 and Additional file 2: Figure S2. The top three hypermethylated loci were cg03110921 (associated with *DLG5*), cg20968678 (associated with *TMEM169*), and cg14087413 (associated with *TMEM8B*). The top three hypomethylated loci were cg18188328 (associated with *JARID2*), cg02522196 (not associated with genes), and cg02291365 (not associated with genes). The dot plots have consistent differential methylation patterns in these CpG sites between preeclampsia cases and controls.

### Genome-scale patterns of differentially methylated CpG sites in association with genes

According to the Illumina 450 K annotation, we categorized the CpG sites in association with genes into seven groups, namely the TSS1500 (200 to 1,500 bp upstream of transcription starting sites), TSS200 (the transcription starting sites to 200 bp upstream interval), 5′ UTR, first exon, gene body, 3′ UTR, and intergenic regions. Significant differences between observed and background frequencies are seen in all the seven CpG categories in association with genes (Figure 3B). As expected from the annotations by CpG island and surrounding areas, the most striking results are the significantly higher percentages of overall DM, hypermethylated and hypomethylated sites in the gene body and intergenic regions, compared to the background percentage of probes located in these regions. Similar results are also observed in the 3′ UTR. These results highlight the significance of non-promoter-associated differential methylation in EOPE. Consistent with the observations in the CpG islands, we find that there is a significantly lower percentage of DM and hypermethylated and hypomethylated sites in the TSS200 and first exon regions compared to their background probe percentages. The hypomethylation pattern in the TSS200 region is also evident in the heatmap results of CpG islands with unsupervised hierarchical clustering, where the case and control groups are clearly separated (Figure 4B). On the other hand, the TSS1500 regions have a significantly lower percentage of hypomethylated sites but about the same percentage of hypermethylated sites compared to background percentages.

### Promoter-associated network and pathway analysis

Given that methylation of CpG islands and promoter regions have received the most attention in determining how epigenetic changes affect gene expression [25,26], we focused the rest of the analysis on the TSS200 region. For each gene, we took the sum of the Beta values and performed a DM test on the summed Beta values, using a moderated *t*-test. As a result, 643 genes with hypomethylated promoters and 389 genes with hypermethylated promoters were identified.

**Figure 5:**
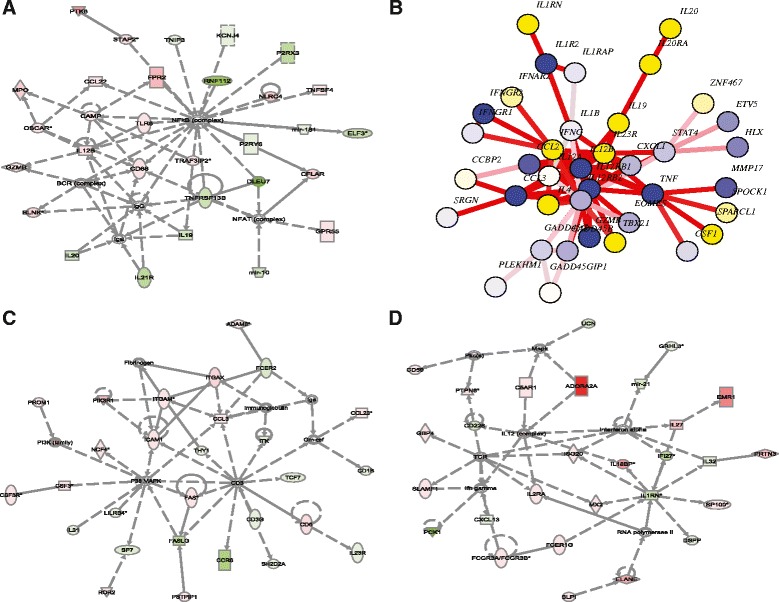
**Top networks in IPA and EpiMods analysis. (A)** IPA network involved in the inflammatory response. **(B)** EpiMod validation network containing genes involved in an immune/inflammatory process. **(C)** IPA network involved in cellular development, growth and proliferation, and hematological system development and function. **(D)** IPA network involved in cell-to-cell signaling, hematological system development and function, and immune cell trafficking. Red and green colors represent hypermethylation and hypomethylation in the promoter of the gene, respectively.

We then performed an Ingenuity Pathway Analysis (IPA) on the genes with significant DM at TSS200 to identify pathways and networks that were altered in EOPE (Figure 5A, C, D, Tables 2 and 3). The top five networks were as follows: 1) inflammatory response, cellular function and maintenance, and respiratory disease; 2) cellular development, cellular growth and proliferation, and hematological system development and function; 3) cell-to-cell signaling and interaction, hematological system development and function, and immune cell trafficking; 4) molecular transport, cellular movement, and hematological system development and function; and 5) cellular development, cellular growth and proliferation, and hematological system development and function. Many genes previously shown to be important in EOPE were found in these top networks, such as *PI3K1*, *FAS*, *IGF1*, and *IL12B*. In addition, several microRNAs appeared in the top five networks, such as *mir-10a*, *mir-181c*, *mir-21*, *mir-101-1*, and *mir-29a*. Interestingly, the top canonical pathways were as follows: (1) FXR/RXR activation, (2) granulocyte adhesion and diapedesis, (3) altered T-cell and B-cell signaling in rheumatoid arthritis, (4) hepatic cholestasis, and (5) agranulocyte adhesion and diapedesis.

**Table 2:** **CpG sites and genes of interest (significant CpG sites of interest)**

**CpG site**	**Case mean**	**Control mean**	***P*** **value (adjusted)**	**Gene**
cg05025071	1.040	−0.338	0.000	*EMR1*
cg02138358	1.432	0.359	0.000	*MPO*
cg16052198	−0.510	−1.522	0.000	*FPR2*
cg03633458	0.977	0.075	0.000	*ELANE*
cg21308575	−0.098	−1.172	0.000	*EMR1*
cg05094429	1.317	2.237	0.000	*CCR6*
cg12230709	0.068	−0.977	0.001	*PRTN3*
cg10161121	0.801	1.612	0.001	*FASLG*
cg06606386	−2.153	−3.251	0.001	*MIR27A*
cg16572540	0.012	−0.640	0.001	*MIR24-2*
cg07055315	0.326	−0.759	0.001	*NLRC4*
cg02515217	−0.141	1.510	0.001	*MIR21*
cg18447740	−0.352	−1.082	0.002	*OSCAR*
cg24964368	−0.331	−1.130	0.002	*FPR2*
cg08198483	−0.049	−0.769	0.002	*MIR24-2*
cg12123019	−0.681	−1.686	0.003	*IL12RB1*
cg17004025	0.052	−0.591	0.005	*PRTN3*
cg06100973	1.445	0.376	0.006	*ELANE*
cg05914150	2.411	1.611	0.006	*PIK3R1*
cg25341726	1.384	0.678	0.007	*IL27*
cg11582579	1.420	2.189	0.007	*IL19*
cg20660269^a^	0.303	−1.105	0.007	*ADORA2A*
cg04626878	−0.621	−1.509	0.013	*PTK6*
cg26149678	−0.592	−1.907	0.013	*IL18BP*
cg25091228	1.637	2.463	0.014	*PIK3R1*
cg10861599	−1.713	−2.283	0.015	*TNFSF4*
cg25756470	−2.031	−2.604	0.016	*NCF4*
cg14916288	−1.137	−1.756	0.022	*CCL23*
cg23763137	−0.032	−1.004	0.025	*ADORA2A*
cg16257983	−2.474	−3.092	0.026	*FAS*
cg11783497	0.522	1.625	0.026	*IL1RN*
cg08491125	−3.161	−3.864	0.027	*ISG20*
cg25247520	0.360	0.939	0.031	*MIR1204*
cg12929678	0.937	1.384	0.032	*CD226*
cg03974286	−0.676	−1.047	0.033	*CFLAR*
cg13747967	−1.309	−1.947	0.037	*CCL22*
cg01436254	0.913	0.439	0.042	*CD86*
cg00590039	−1.184	−1.738	0.043	*TRAF3IP2*
cg11201447	−0.109	0.567	0.043	*MIR1204*
cg13904968	0.567	1.788	0.044	*PCK1*
cg26394055	−0.293	−0.927	0.045	*FCER1G*

^a^This CpG site is significantly hypermethylated by comparing to the results of Cruickshank *et al.* [32].

**Table 3:** **CpG sites and genes of interest (genes with significant TSS200)**

**Gene**	**Case**	**Control**	**IPA network**
*CCL22*	0.164	0.096	Inflammatory response, cellular function and maintenance, respiratory disease
*CD86*	1.014	0.875	Inflammatory response, cellular function and maintenance, respiratory disease
*CFLAR*	1.366	1.161	Inflammatory response, cellular function and maintenance, respiratory disease
*FPR2*	0.638	0.363	Inflammatory response, cellular function and maintenance, respiratory disease
*IL19*	1.545	1.727	Inflammatory response, cellular function and maintenance, respiratory disease
*MPO*	0.588	0.388	Inflammatory response, cellular function and maintenance, respiratory disease
*NLRC4*	0.696	0.471	Inflammatory response, cellular function and maintenance, respiratory disease
*OSCAR*	1.687	1.135	Inflammatory response, cellular function and maintenance, respiratory disease
*PTK6*	2.161	1.752	Inflammatory response, cellular function and maintenance, respiratory disease
*TNFSF4*	0.254	0.187	Inflammatory response, cellular function and maintenance, respiratory disease
*TRAF3IP2*	3.943	3.651	Inflammatory response, cellular function and maintenance, respiratory disease
*CCL23*	0.655	0.477	Cellular development, cellular growth and proliferation, hematological system development and function
*CCR6*	3.000	3.299	Cellular development, cellular growth and proliferation, hematological system development and function
*FAS*	1.226	0.865	Cellular development, cellular growth and proliferation, hematological system development and function
*FASLG*	1.294	1.566	Cellular development, cellular growth and proliferation, hematological system development and function
*NCF4*	0.766	0.532	Cellular development, cellular growth and proliferation, hematological system development and function
*PIK3R1*	3.526	3.337	Cellular development, cellular growth and proliferation, hematological system development and function
*ADORA2A* ^a^	1.879	1.077	Cell-to-cell signaling and interaction, hematological system development and function, immune cell trafficking
*CD226*	1.384	1.554	Cell-to-cell signaling and interaction, hematological system development and function, immune cell trafficking
*ELANE*	1.190	0.876	Cell-to-cell signaling and interaction, hematological system development and function, immune cell trafficking
*EMR1*	1.146	0.699	Cell-to-cell signaling and interaction, hematological system development and function, immune cell trafficking
*FCER1G*	0.176	0.105	Cell-to-cell signaling and interaction, hematological system development and function, immune cell trafficking
*IL27*	1.105	0.856	Cell-to-cell signaling and interaction, hematological system development and function, immune cell trafficking
*IL18BP*	5.765	4.816	Cell-to-cell signaling and interaction, hematological system development and function, immune cell trafficking
*IL1RN*	1.998	2.652	Cell-to-cell signaling and interaction, hematological system development and function, immune cell trafficking
*ISG20*	0.140	0.079	Cell-to-cell signaling and interaction, hematological system development and function, immune cell trafficking
*PCK1*	1.725	2.310	Cell-to-cell signaling and interaction, hematological system development and function, immune cell trafficking
*PRTN3*	0.911	0.637	Cell-to-cell signaling and interaction, hematological system development and function, immune cell trafficking
*MIR24-2*	1.044	0.801	Inflammatory response, cellular development, tissue development
*MIR1204*	1.451	1.630	Inflammatory response, cellular development, tissue development
*MIR21*	1.736	1.911	Cell-to-cell signaling and interaction, hematological system development and function, immune cell trafficking
*MIR27A*	0.279	0.217	Inflammatory response, cellular development, tissue development

^a^This gene has significant methylation changes in the TSS200 related to gestational age, by comparing to the results of Cruickshank *et al.* [32].

We next used the software package EpiMod to validate the network findings from IPA. Previously, EpiMod was used to identify DM hotspots in the Illumina Infinium 27 K platform, by integrating methylation data with protein interaction networks using the methylation of promoter-associated CpG sites [26]. We have extended the package to include promoter-associated probes from Illumina 450 K. Similarly to the IPA analysis, we used the sum of Beta values within the TSS200 as the measure for DM, in order to perform the EpiMod analysis. Many key genes in the EpiMod networks were also hubs in the IPA network analysis, and the networks themselves were associated with many of the same functions. We show one exemplary module from EpiMods with the hotspot genes *IL12B* and receptors *IL12RB1*/*B2* (Figure 5B). Similar to the best ranked network in IPA (Figure 5A), this epi-module shows a clear enrichment of genes involved in the inflammatory response. Consistently, the Kyoto Encyclopedia of Genes and Genomes (KEGG) pathway enrichment analysis on the genes found in this epi-module shows that “Cytokine-cytokine receptor interaction” is the most significant pathway (FDR = 2.5e − 21).

### Differential methylation in the non-coding genomic regions

Since more than 95% of the genome does not encode proteins, we next examined the CpG sites in non-coding RNAs. We first analyzed microRNAs, given that they are relatively better understood compared to other non-coding RNAs (ncRNAs). The microRNAs present in the top ten IPA networks were grouped based on their methylation status (hypo- vs. hypermethylation), and their targets were predicted with the TargetScan program. This list of target genes was then subjected to KEGG analysis using the DAVID online functional annotation tool [27]. Pathways with a BH-adjusted *P* value less than 0.05 were considered significant. The pathways that were enriched in the hypomethylated and hypermethylated microRNA target gene sets were similar, with common signaling pathways such as the “ErbB signaling pathway” and the “neurotrophin signaling pathway” (Table 4). Other common biological processes such as “endocytosis,” as well as diseases such as “Chronic myeloid leukemia” were also enriched.

**Table 4:** **Enriched KEGG pathways from predicted targets of top DM microRNAs**

	**Count**	**Percent of genes**	**Fold enrichment**	***P*** **value (FDR)**
Predicted targeted pathways of hypomethylated miRs				
Glioma	45	6.42%	1.9269	0.0001
Axon guidance	77	10.99%	1.6102	0.0003
Pathways in cancer	166	23.69%	1.3653	0.0004
Endocytosis	101	14.41%	1.4808	0.0011
Insulin-signaling pathway	77	10.99%	1.5386	0.0040
ErbB-signaling pathway	54	7.71%	1.6744	0.0056
Neurotrophin-signaling pathway	71	10.13%	1.5446	0.0086
Chronic myeloid leukemia	47	6.71%	1.6905	0.0185
SNARE interactions in vesicular transport	28	4.00%	1.9877	0.0253
Predicted targeted pathways of hypermethylated miRs				
Endocytosis	133	14.32%	1.4482	0.0000
MAPK-signaling pathway	178	19.17%	1.3357	0.0000
Neurotrophin-signaling pathway	92	9.91%	1.4865	0.0001
Axon guidance	95	10.23%	1.4755	0.0001
Pathways in cancer	211	22.73%	1.2889	0.0001
Chronic myeloid leukemia	59	6.35%	1.5761	0.0011
ErbB-signaling pathway	65	7.00%	1.4969	0.0061
Apoptosis	65	7.00%	1.4969	0.0061
Regulation of actin cytoskeleton	140	15.08%	1.3046	0.0074
Adherens junction	58	6.25%	1.5092	0.0147
Glioma	49	5.28%	1.5583	0.0196
Focal adhesion	130	14.00%	1.2958	0.0275

*SNARE* soluble NSF attachment protein receptor, *MAPK* mitogen-activated protein kinase.

We next predicted the function of various long intergenic non-coding RNAs (lincRNAs) that had DM in TSS200 regions. In order to represent the protein-coding genes with significant DM at the pathway level, the summed β-values of the TSS200 regions of DM protein-coding genes were used to generate a pathway-based deregulation score matrix (PDS matrix). This matrix is a measure of how much the activities of various pathways deviate from normal. The summed β-values of the TSS200 regions of DM lincRNAs were then correlated with the scores in the PDS matrix using the Spearman correlation coefficient (*ρ*). Only lincRNA-pathway pairs that satisfied the cutoff absolute (*ρ*) >0.90 and *P* <0.05 are considered significant. A total of 96 KEGG and BIOCARTA pathways stand out, many of which are related to the immune response and inflammation [see Additional file 3: Table S2].

### Tissue differences and genes of interest

We previously reported a global hypermethylation coupled with hypomethylation in promoters from the chorioamniotic membrane of EOPE patients [28]. Some of the patients who participated in that study gave cord blood samples for this study. To determine the contribution of tissue specificity vs. disease (EOPE) specificity, we compared the subsets of methylation data from the coupled chorioamniotic membrane and cord blood samples. An ANOVA model containing terms for preeclampsia and the tissue type was used to gain insight on their contributions. The resulting F-statistics show that tissue type rather than EOPE is the dominant factor responsible for the variance among the methylation data [see Additional file 4: Figure S3].

We further analyzed the DM sites in both chorioamniotic membranes and the cord blood, with moderated *t*-tests subject to BH multiple hypothesis testing (MHT). The analysis revealed that 10,510 CpG sites were considered DM, and among them, 10,355 CpG sites were consistently hypermethylated or hypomethylated in both tissues. These sites were mapped to the TSS200 regions of 579 genes. Multiple genes from the IPA and EpiMod networks were found among these 579 genes, including the highly connected genes *CD86*, *IL19*, *OSCAR*, *TRAF3IP2*, *FAS*, *PIK3R1*, *IFNG*, *IL1RN*, and *ISG20*. These results suggest that a small number of genes involved in inflammation and lipid metabolism are epigenetically modified similarly in both membrane tissues and cord blood of fetal origin.

## Discussion

### Comparison with other cord blood methylation studies of preeclampsia

Compared to the number of methylation studies in placenta and maternal peripheral blood tissues related to EOPE, there are surprisingly few reports on umbilical cord blood DNAs. This might be partially due to the fact that EOPE is a maternal disease. However, given the well-established link between preterm birth and adulthood onset diseases such as hypertension, cardiovascular diseases, and obesity, it is plausible that some long-term consequences are carried through the changes made by “epigenetics markers” at or even before birth. In this study, we describe in depth the significant genome-scale hypomethylation observed in cord blood DNAs associated with maternal EOPE, based on the Illumina 450 K. Our study confirms and expands on the very few earlier methylation studies of cord blood samples from preterm deliveries (or more specifically, preterm deliveries due to EOPE), which had either lower throughput or lacked gene level resolution for DNA methylation. Burris *et al.* used LINE-1 methylation levels as a surrogate measurement of the global methylation and found that after adjustment for other confounding factors, a lower cord blood LINE-1 level methylation was associated with a shorter gestation (−0.45 weeks, (95% CI −0.83, −0.08)) and higher odds of preterm birth (OR 4.55 (95% CI 1.18, 17.5)) [23]. On the other hand, He *et al.* conducted a pilot study to examine DNA methylation levels of *IGF2* and *GNAS* in cord blood associated with preeclampsia. They found that the average methylation level of *IGF2* but not *GNAS* was significantly lower in the preeclampsia samples [21]. We used a much higher throughput method with the Illumina 450 K and also found that *IGF2* has one CpG site with a significantly lower methylation level in the gene body (cg01668279, FDR adjusted *P =* 0.0429). However, unlike the other study, we found that seven out of nine CpG sites associated with *GNAS* have significantly lower methylation in EOPE, in regions ranging from the TSS1500 to the gene body. The overall agreement between our study and the majority of other studies suggest that EOPE has broad effects resulting in abnormal DNA methylation even in newborn cord blood cells.

### Potential complications in the study due to gestational age and birth weight

In our current experimental design of comparing EOPE vs. full-term delivery, earlier gestational age and lower mean birth weights are naturally coupled with EOPE. We considered EOPE as the major contributing factor of DM, because the majority of the best-fit models have EOPE as the only effective predictor. Also, the inclusion of highly correlated factors in the statistical model, such as gestational age and birth weight that are coupled with EOPE, can inflate the standard error [29] and potentially mask the causative effect of EOPE. Admittedly, these two factors may confound the observed differences due to EOPE. Hence, we compared our results with other studies on the effect of gestational age on placental or cord blood DNA methylation pattern without preeclampsia [30-32]. Studies using cord blood show little evidence of changes in methylation levels within the third trimester [33,34]. In one study, Michels *et al.* found less than 1% of effect comparing cord blood methylation of preterm babies to full-term babies [33]. In another study, Nomura *et al.* found no significant effect on methylation due to gestational age, in both placenta and cord blood (*P =* 0.31 and *P =* 0.82, respectively) [34]. In the placenta, the DNA methylation pattern does change as gestation progresses. However, the major changes of methylation occur from the first to the second trimesters and considerably fewer changes happen after the second trimester [31]. Moreover, within the third trimester, the placental methylation patterns from babies delivered earlier (around 34 weeks) and those of full-term babies are clustered together and they cannot be distinguished. This suggests that the gestational ages within the last trimester have much less effect on the placental DNA methylation. Novakovic *et al.* also stated that the variation among samples in the third trimester was primarily due to environmentally induced or stochastic reasons. Altogether, these studies suggest that very small or minimal differences in the observed methylation patterns are due to gestational age in our study. This is confirmed by Cruickshank *et al.* who found that only 1,555 DM sites from neonatal blood spots were statistically significantly associated with gestational age [32], whereas we found 64,049 DM sites associated with EOPE after MHT correction. Further, we compared the DM sites in both studies and found that only 319 sites are commonly hypomethylated and 139 sites are commonly hypermethylated [see Additional file 5: Table S3]. In summary, we conclude that gestational age and birth weight have insignificant effect compared to the disease of EOPE.

### Methylation changes related to inflammation

Evidence of changes in CpG methylation related to the inflammatory process is strong in our data. In the top-ranked IPA network (Figure 4A) and EpiMod module (Figure 4B), we see that a large number of DM genes (for example, *IL12B*, *IL19*, *IL20*, *CCL2*) are connected to the nuclear factor kappa-light-chain-enhancer of activated B cells (NF-κB) complex, although NF-κB itself is not differentially methylated. Although it is well known that increased inflammatory stress exists in preeclampsia placentas [35,36] and maternal tissues [37], little is known about immunological responses in newborns. Some studies have shown that Interleukin-6 (*IL6*) was significantly elevated in the cord blood from neonates born small with intrauterine growth retardation. However, no direct measurements of *IL6* were made from those born to preeclamptic mothers [38]. In another study, the authors found evidence of higher NKp30 levels in NK cells, but not in the monocytes of cord blood from preeclamptic pregnancies, suggesting that inflammatory responses may be cell-type specific [39]. Since we used the extracted genomic DNA from cord blood cells without the purification of a subpopulation, the subpopulation-specific inflammation response in EOPE could not be studied. However, we do know that the DNA methylation changes occur in many genes involved in inflammation. Meanwhile, we also observe that some methylation differences are very interesting. For example, the hyper-methylation of IL12B is validated by our pyrosequencing, although the Beta value only decreases by 0.1 from 0.3 in cases (*P <* 0.05) (Figure 2E). Corresponding to this finding, others reported that preeclampsia patients had significantly less IL-12 in villous trophoblasts [30], as well as significantly less IL-12 secretion from the decidua [40]. Moreover, a positive correlation between IL-12 and fetal birth weight in the severe preeclamptic group was observed [40]. All the information seems to support the hypothesis that IL12B is repressed at the gene expression level in the cord blood cells. It will be very interesting to validate the IL12B gene expression in fresh cord blood tissue.

### Methylation changes related to potential cardiovascular problems

Interestingly, we found that FXR/RXR activation and hepatic cholestasis are among the top enriched pathways in the IPA analysis of TSS200 DM regions. An important target of FXR/RXR is fatty acid synthase, *FAS* [41], which was consistently hypermethylated in both cord blood and the placental chorioamniotic membrane. We propose two hypotheses that could explain the methylation changes occurring in these pathways. One hypothesis arises from the fact that fetal liver is the main source of hemopoietic cells during the majority of the gestational period [42]; thus, the DM related to FXR/RXR activation and cholestasis in the cord blood cells likely reflects the dysfunction of liver tissues of newborns associated with maternal EOPE. The nuclear receptors farnesoid X receptor (FXR) and liver X receptor (LXR) form heterodimers with the retinoid X receptors (RXRs) to regulate cholesterol and lipid metabolism, and the primary tissue responsible for cholesterol metabolism is the liver, where cholesterol biosynthesis occurs and cholesterol low-density lipoprotein (LDL) is taken up from the plasma by the LDL receptor. Another hypothesis is that the DM observed in the FXR/RXR pathway from the cord blood cells may be attributed to circulating macrophages within the cord blood cells. LXR/RXR dimers in macrophages activate ABC transporters, such as *ABCA1* and *ABCG1*, which efflux cholesterol from macrophages and prevent toxicity due to cholesterol accumulation [43]. In adults, macrophages overloaded with cholesterol (or foam cells), are hallmarks of atherosclerosis [44]. Regardless of the mechanism leading to the DM among genes related to FXR/RXR activation in cord blood cells, the FXR/RXR pathway is known to play an important role in maintaining cholesterol homeostasis. Abnormal methylation in this pathway might be indicative of long-term risks for coronary heart disease due to lipid metabolism dysfunction. Other processes, such as the inflammatory stress mentioned earlier, might also mediate or contribute to higher risks of cardiovascular diseases later in life for babies born to preeclamptic mothers.

### Future work

The samples in this study were obtained from the pre-processed genomic DNA of cord blood cells stored in the biorepository. As no cord blood serum was stored, we could not obtain inflammatory molecule signals or the lipid profiles in the cord blood. We plan to follow up with another human study to validate the candidate biomarker findings in the protein-coding genes and noncoding RNAs at the methylation level and the expression level.

## Conclusions

To our knowledge, this study is the first to address the genome-scale methylation changes in the cord blood cells associated with EOPE patients. The newborn cord blood cells are thought to originate from the fetal liver; thus, much of the methylation changes involve signatures of liver functions. We have found significant genome-scale DNA methylation modifications that may suggest a link between inflammation and lipid dysregulation in newborns. These findings may suggest a link between maternal EOPE and newborns with an increased lifetime risk of cardiovascular diseases.

## Methods

### Patient specimens

We utilized samples from the RMATRIX Hawaii Biorepository (HiBR), which has obtained its own IRB approval. From this repository, a total of 21 cord blood samples were initially identified. Maternal cases had EOPE at <34 weeks gestation and controls were full-term pregnancies without preeclampsia. To ensure the quality of the cord blood samples, maternal patients with known medical or obstetrical disorders associated with preeclampsia were excluded, including diabetes mellitus or gestational diabetes mellitus, hypertension, renal disease, hyperthyroidism, and systemic lupus erythematous. Only singleton pregnancies were included.

### DNA isolation

Genomic DNA from whole cord blood cells was extracted by the HiBR laboratory. Clotted cord blood was centrifuged through a Clotspin Basket (Qiagen) at 2,000 × *g* for 5 min to disperse the clot. Three volumes of Red Blood Cell (RBC) Lysis Solution (Qiagen) were added to one volume of dispersed cord blood. The samples were briefly vortexed, shaken for 15 min at room temperature then centrifuged as above to pellet leukocytes and clot particulates. The pellet was resuspended in Cell Lysis Solution (Qiagen) containing Proteinase K (Qiagen) at 20 mg/ml and then incubated at 55°C overnight. The Gentra Systems Autopure LS (Qiagen) was used to extract the DNA from the sample using the Compromised Cell Lysate Protocol. The purified DNA solution was then incubated in a 65°C water bath for 1 h and shaken at room temperature overnight.

### Genome-scale methylome profiling using the HumanMethylation450 BeadChip

Methylation profiling was done using the Illumina Infinium methylation assay and the HumanMethylation450 BeadChip. First, the cord blood DNA samples were pre-screened for the suitability of the Methylation450 assay using Nanodrop to check the purity and the PicoGreen assay to check the dsDNA concentration. The EZ DNA Methylation kit was used for the bisulfite conversion (Zymo Research), and 100 to 250 ng of bisulfite-converted DNA was hybridized onto the Infinium Human Methylation450 BeadChip following the Illumina Infinium HD Methylation protocol.

### Pyrosequencing validation

Primer design was carried out using the PyroMark Assay Design 2.0 software. One of the primers was biotinylated to enable capture by Streptavidin Sepharose. Bisulfite-treated DNA was amplified followed by pyrosequencing using the PyroMark PCR kit (Qiagen) on a PyroMark Q24 instrument using PyroMark Gold Q24 Reagents, according to the manufacturer’s protocol. The primers for the seven CpG sites selected for validation are listed in Additional file 6: Table S1.

### Methylation data preprocessing

Raw data preprocessing was performed using the R package “minfi” [45]. The background signal was subtracted from the raw intensity data, and any probes with a detection *P* value greater than 0.05 were eliminated. The intensity value for each probe was transformed first to methylation Beta values and then to M-values (the logit transform of Beta values) and normalized using the SWAN algorithm [46]. Probes with known SNPs based on the latest annotation by Illumina were removed from downstream analysis due to the concern that they could confound intensity changes during later analysis steps. Probes for CpG sites on sex chromosomes were removed to eliminate the effect of the genders of newborns.

### Site-level differential methylation (DM) analysis

Before DM analysis, samples were examined for the differences using the primary physiological or clinical parameters. As a result, 12 cases (one case was eliminated due to the outlier birth weight) and eight controls were determined suitable for DM. The AIC was used to select the primary physiological or clinical parameters in a linear model that best explains the differences among the methylation data. The model that optimized the AIC score most frequently was the model with “preeclampsia” as the only contributing variable. The importance of this term was verified using ANOVA analysis, where the preeclampsia factor was found to have the largest value for its average F-statistic. A moderated *t*-test from the “limma” package was used to determine the statistical significance of difference in methylation for each CpG site between cases and controls. The resulting *P* value was then adjusted for MHT using the BH correction. CpG sites with an adjusted *P* value less than 0.05 were considered significant. Correlation heatmaps, PCA plots, volcano plots, and bar graphs, and so on were generated using R 3.0.2.

### Ingenuity pathway analysis (IPA)

Since the promoter region plays a critical role in transcriptional regulation, the sum of the Beta values of CpG sites in the TSS200 region was used as a proxy for gene methylation levels. The “limma” package’s moderated *t*-test was used to determine the statistical significance of methylation differences between cases and controls for each gene. Similar to the site-level analysis, *P* values were corrected for MHT using the BH correction, and adjusted *P* values less than 0.05 were considered significant. The log2-fold change in methylation values was calculated, and values for significantly differentially methylated genes were used as inputs to IPA to generate gene interaction networks and canonical pathways that are significantly associated with EOPE.

### EpiMod analysis

Similar to the IPA analysis, the sums of Beta values for sites in TSS200 regions were used as inputs for the functions in the EpiMod package [26]. The EpiMod package was originally developed to analyze Illumina 27 K data. We modified the code to analyze 450 K data by replacing the methylation values of each gene with the sum of TSS200, similar to the newer package Functional Epigenetic Modules (FEM) from the same developers of EpiMods [47].

### Non-coding RNA methylation analysis

Two types of ncRNA were analyzed: microRNA and lincRNA. TargetScan was used to predict mRNA targets for the microRNAs that were found in the top ten IPA networks. The microRNA targets were then subjected to GO and KEGG analysis to determine the enriched gene sets among the predicted targets. For lincRNA analysis, the CpG sites were associated with annotated lincRNAs from the UCSC browser, and lincRNAs were tested for differential methylation analysis in the TSS200 regions, similar to the approach used for the protein-coding genes described earlier (see the “Methods” section for IPA analysis). To predict the function of differentially methylated lincRNAs, the “pathifier” package was used to create a PDS matrix [48], using the DM protein-coding RefSeq genes per their sum of β-values in the TSS200 region. Spearman’s rho (*ρ*) correlation coefficient was used to calculate the correlation between the lincRNA methylation and the pathways built from DM RefSeq genes in the samples. Pathway-lincRNA pairs with |*ρ*| > 0.90 were kept for functional analysis.

## Additional files

Additional file 1: Figure S1. Top 20 hypomethylated CpG sites. Dot plots of statistically significant top 20 hypomethylated CpG sites with Beta value difference greater than 0.2. Additional file 2: Figure S2. Top 20 hypermethylated CpG sites. Dot plots of statistically significant top 20 hypermethylated CpG sites. Additional file 3: Table S2. LincRNA pathway analysis. KEGG and BIOCARTA pathways with high correlations (*ρ* > 0.90) and significant *P* values (*P <* 0.05) for lincRNAs. Additional file 4: Figure S3. ANOVA plot of two factor analysis. Average F-statistics plot of tissue and disease factors using the summed TSS200 methylation Beta values in cord blood and paired chorioamniotic membrane samples. Additional file 5: Table S3. CpG sites related to preeclampsia and gestational age. A list of CpG from a comparison of the differentially methylated CpG sites in preeclampsia with the results of Cruickshank *et al.* [32] on gestational age. Additional file 6: Table S1. Pyrosequencing primers. A list of the primer sequences used to validate important CpG sites.

## Abbreviations

AIC: Akaike’s information criterion
ANOVA: Benjamini-Hochberg (BH) correction, analysis of variance
DM: differential methylation or differentially methylated
EOPE: early onset preeclampsia
HiBR: RMATRIX Hawaii Biorepository
IPA: ingenuity pathway analysis
LOPE: late onset preeclampsia
MHT: multiple hypothesis testing
