# Supplementary figures and images for: Genome-scale hypomethylation in the cord blood DNAs associated with early onset preeclampsia
Source: Clin Epigenetics. 2015 Mar 13;7(1):21. doi: 10.1186/s13148-015-0052-x (PMC4371797; doi:10.1186/s13148-015-0052-x)

**Figure S1**

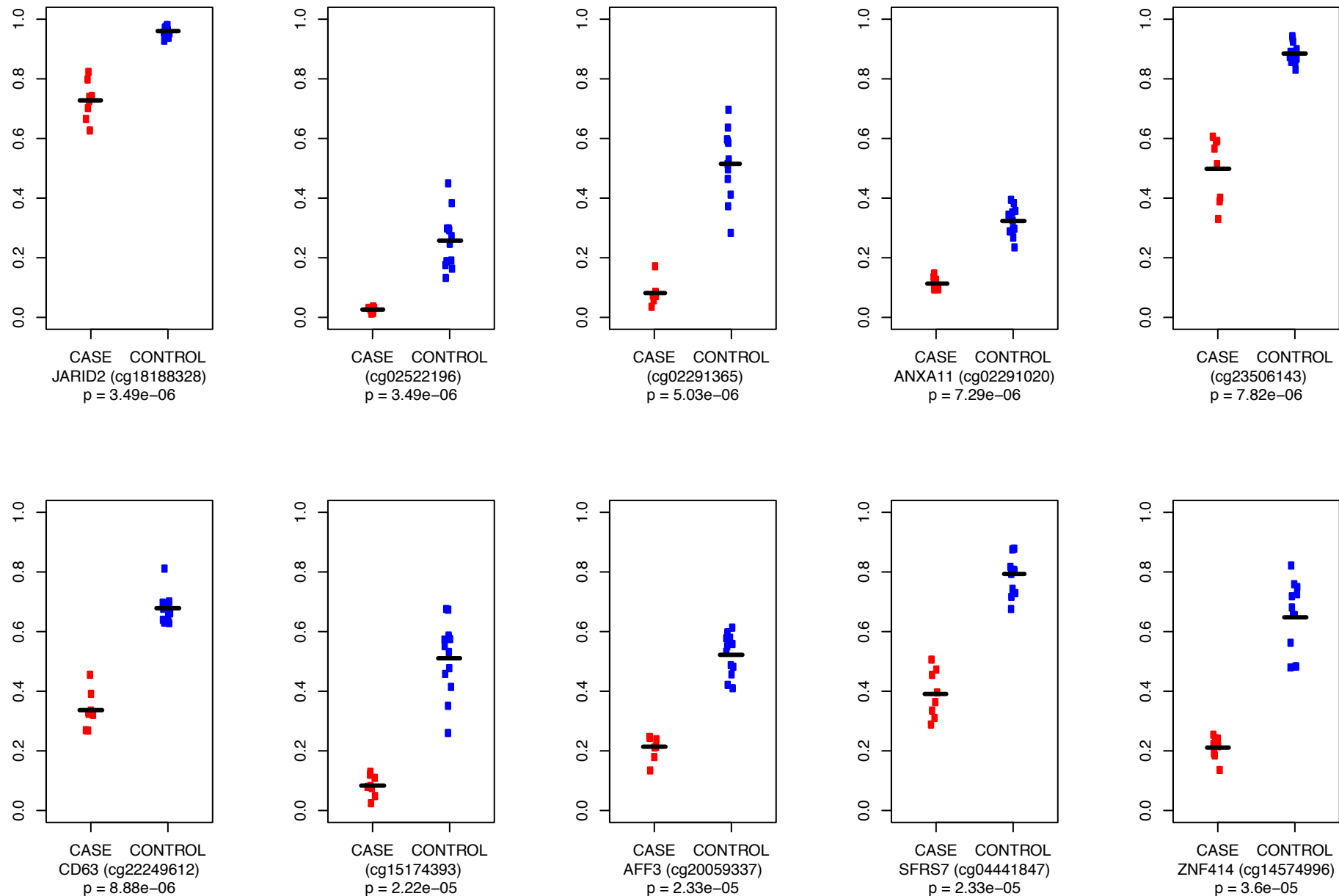

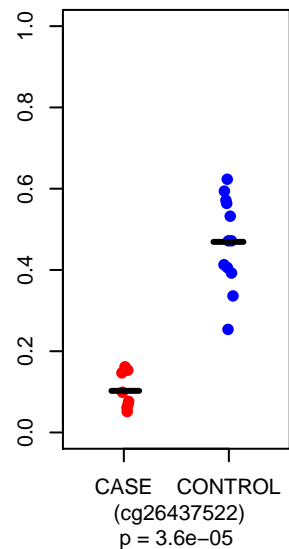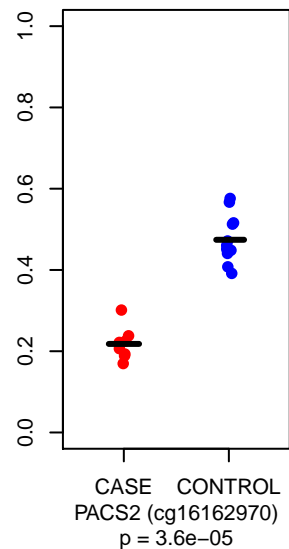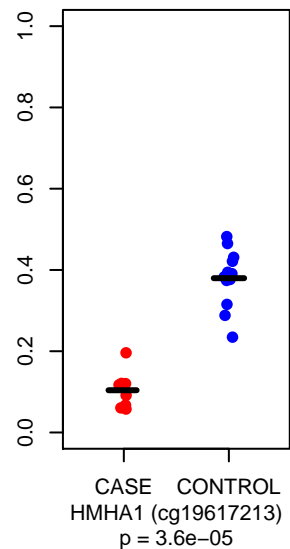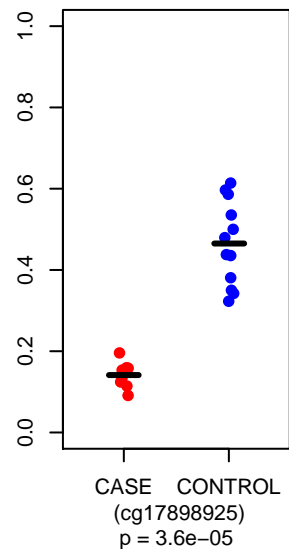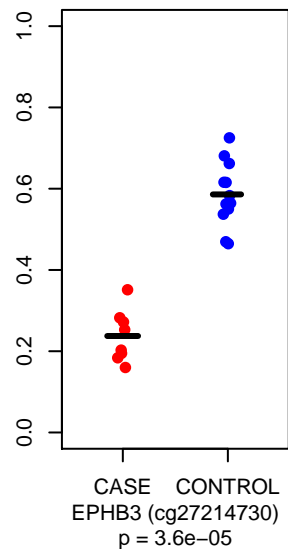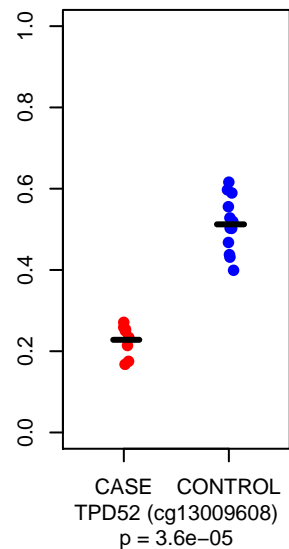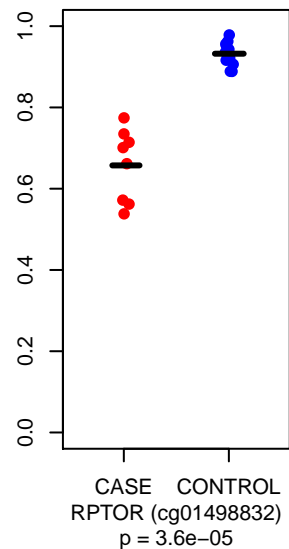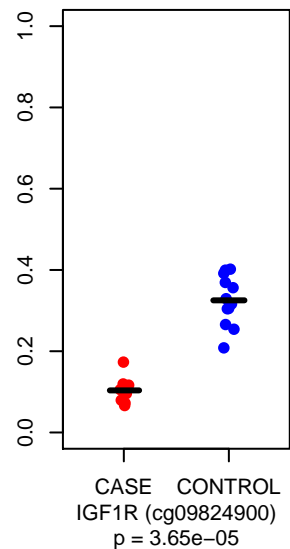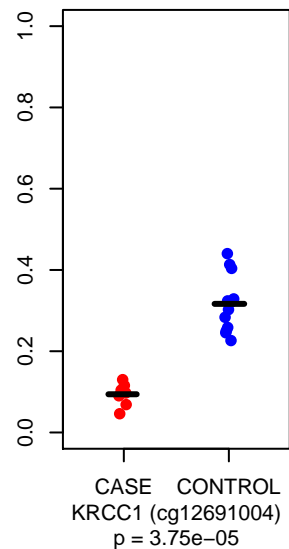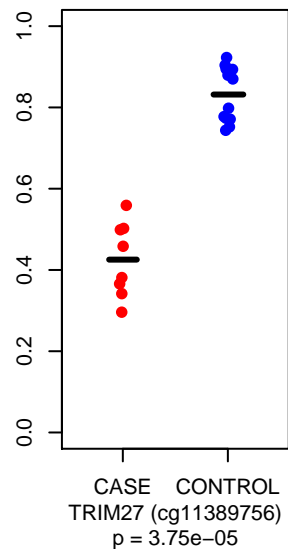

Supplement: Additional file 1: Figure S1. — Top 20 hypomethylated CpG sites. Dot plots of statistically significant top 20 hypomethylated CpG sites with Beta value difference greater than 0.2. [file 13148_2015_52_MOESM1_ESM.pdf]

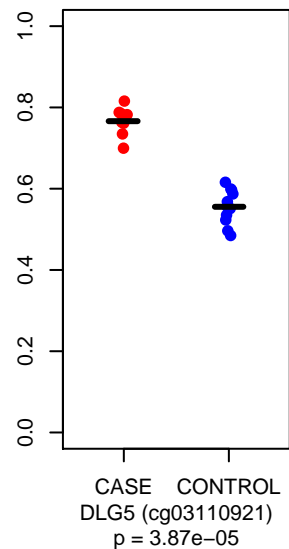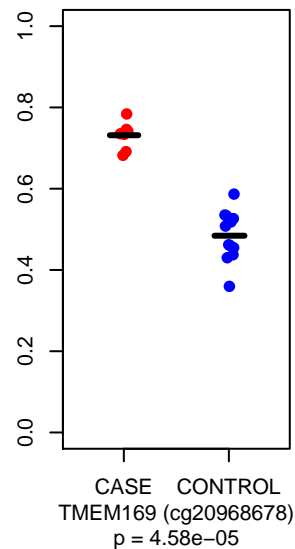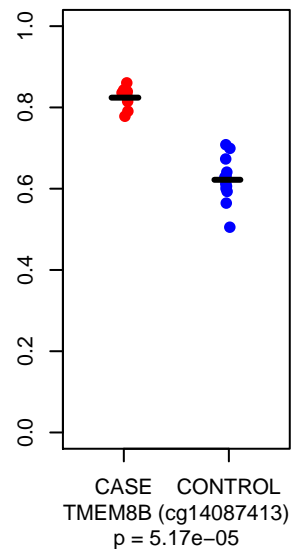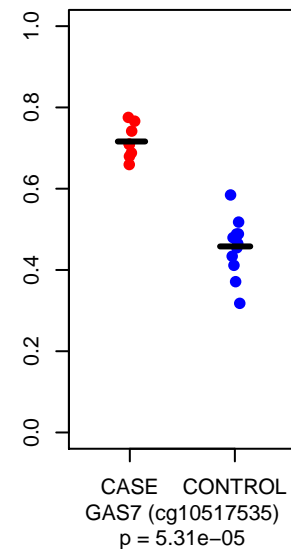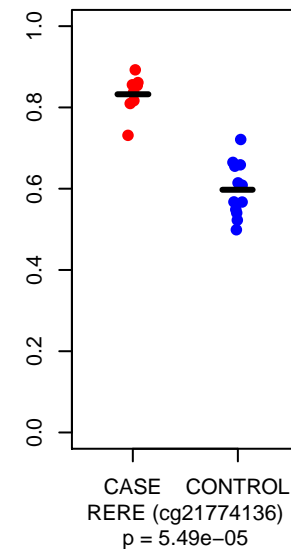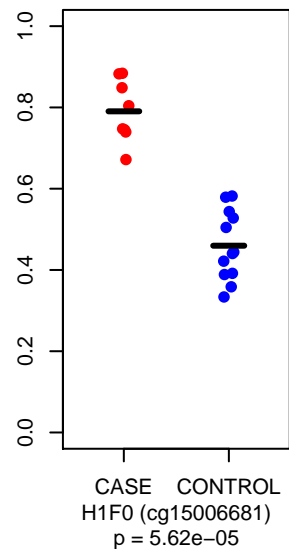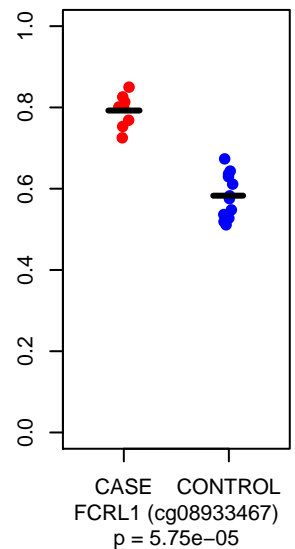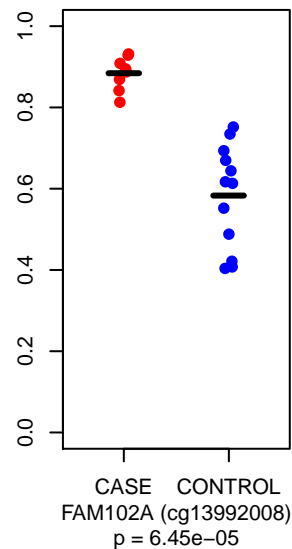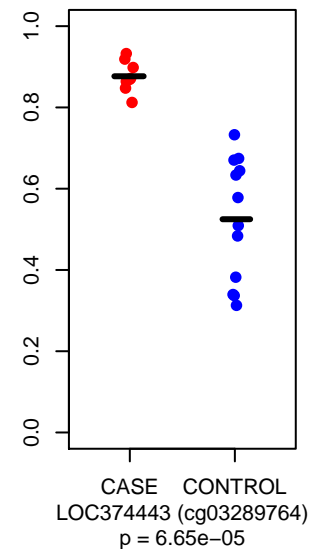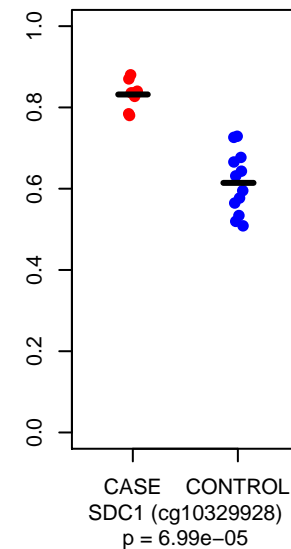

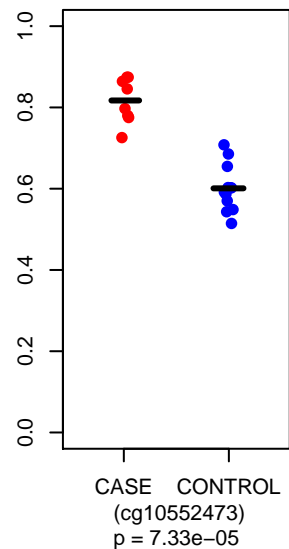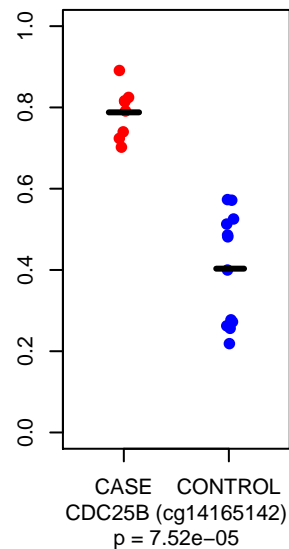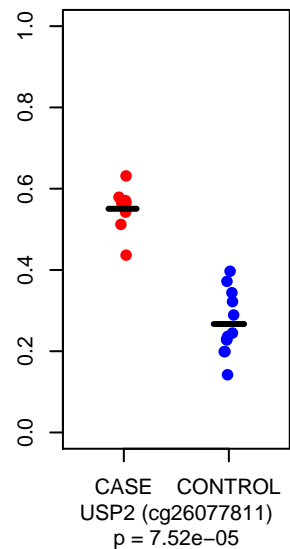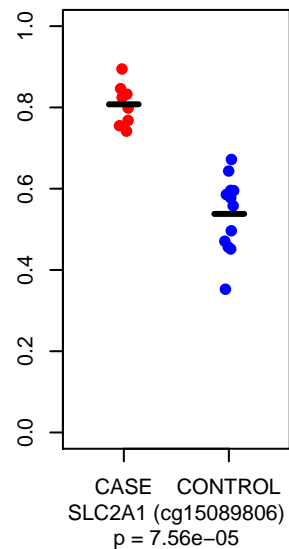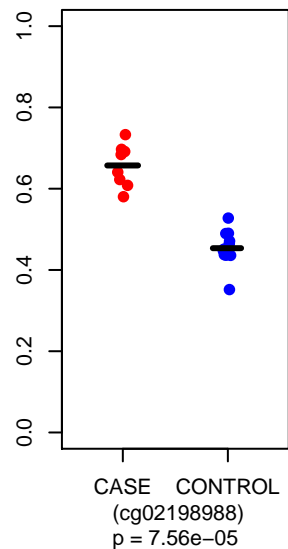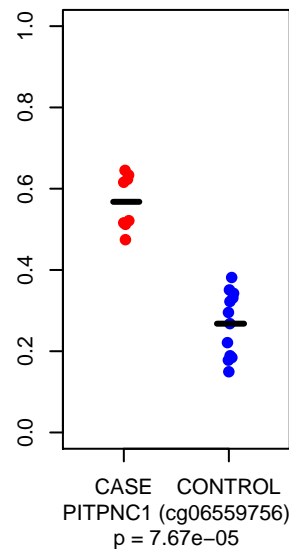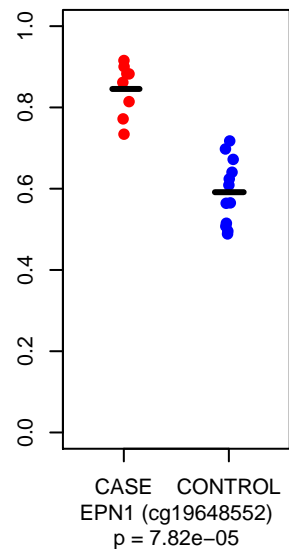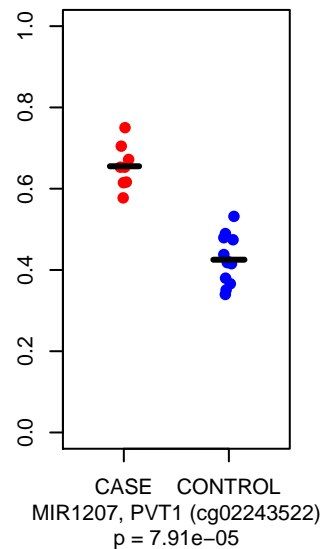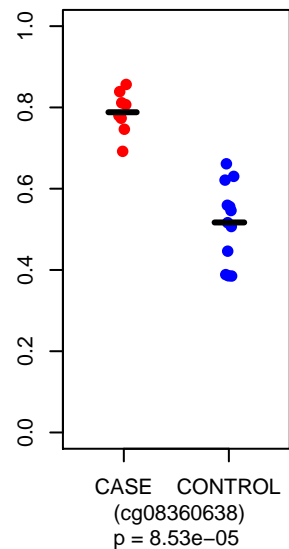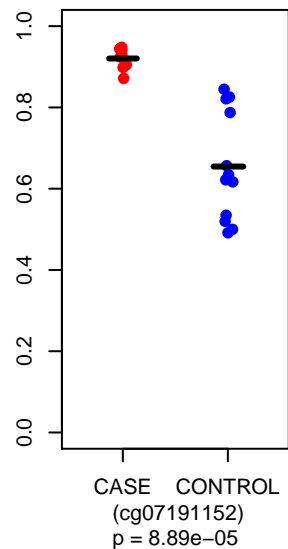

Supplement: Additional file 2: Figure S2. — Top 20 hypermethylated CpG sites. Dot plots of statistically significant top 20 hypermethylated CpG sites. [file 13148_2015_52_MOESM2_ESM.pdf]

**Figure S3**

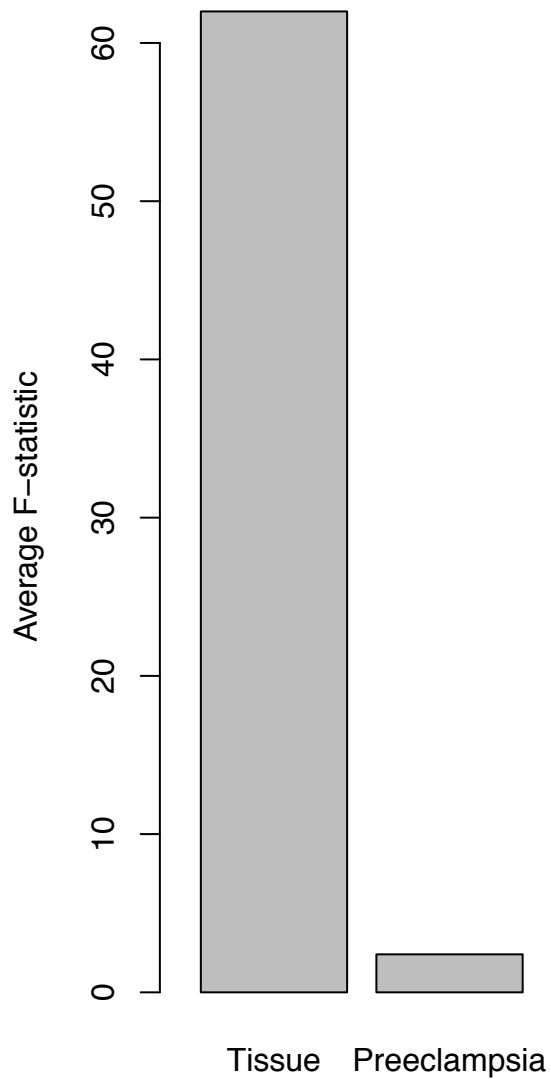

Supplement: Additional file 4: Figure S3. — ANOVA plot of two factor analysis. Average F-statistics plot of tissue and disease factors using the summed TSS200 methylation Beta values in cord blood and paired chorioamniotic membrane samples. [file 13148_2015_52_MOESM4_ESM.pdf]
